# Augmenting Patient Education in Hand Surgery—Evaluation of ChatGPT as an Informational Tool in Carpal Tunnel Syndrome

**DOI:** 10.3390/medicina61091677

**Published:** 2025-09-16

**Authors:** Benedikt Fuchs, Nikolaus Thierfelder, Irene Mesas Aranda, Verena Alt, Constanze Kuhlmann, Elisabeth M. Haas-Lützenberger, Konstantin C. Koban, Riccardo E. Giunta, Sinan Mert

**Affiliations:** Division of Hand, Plastic and Aesthetic Surgery, LMU University Hospital, Munich 80336, Germanyverena.alt@med.uni-muenchen.de (V.A.); elisabeth.haas@med.uni-muenchen.de (E.M.H.-L.); riccardo.giunta@med.uni-muenchen.de (R.E.G.);

**Keywords:** carpal tunnel syndrome, patient education, ChatGPT, large language models, artificial intelligence

## Abstract

*Background and Objectives*: Carpal tunnel syndrome (CTS) is the most common entrapment neuropathy caused by chronic compression of the median nerve within the carpal tunnel. Patient education is a cornerstone of informed consent and postoperative outcomes, yet time constraints often limit traditional consultations. Recently, generative AI tools such as ChatGPT have emerged as potential adjuncts in delivering standardized medical information. *Materials and Methods*: This study evaluated the quality and comprehensiveness of ChatGPT-generated patient education on CTS and open carpal tunnel release. A standardized prompt was used with ChatGPT-4o to generate educational material. A structured and standardized questionnaire was then administered to both patients and physicians (*n* = 8) to assess content quality, clarity, comprehensiveness, and perceived usefulness. *Results*: Both patients and physicians reported high satisfaction with the information provided. The etiology, procedural risks, and general anatomical principles were well conveyed. However, certain intraoperative concepts—such as neurolysis, synovectomy, and hemostasis—were underrepresented. While conservative therapies were addressed, the omission of endoscopic surgical options limited informational completeness. Prognostic information and long-term consequences of untreated CTS were rated as average by some participants. Postoperative guidance was adequately covered but lacked individualized nuance. *Conclusions*: ChatGPT shows promise as an adjunct in surgical patient education, offering clear and standardized information. Nevertheless, it is not a substitute for clinician–patient interaction. While it may bridge preliminary knowledge gaps, emotional support and individualized consent discussions remain essential. Further refinement and clinical validation of AI-generated educational content are needed to ensure safe and effective integration into routine practice.

## 1. Introduction

Carpal tunnel syndrome (CTS) is a common nerve entrapment disorder caused by compression of the median nerve beneath the flexor retinaculum, often affecting women aged 40–50 [1]. Symptoms include nocturnal pain, paresthesia, and, in advanced stages, thenar atrophy [1,2]. Diagnosis was confirmed from clinical examination and electroneurography. Mild cases may benefit from splinting and NSAIDs (non-steroidal anti-inflammatory drugs), while surgery—open or endoscopic release of the flexor retinaculum—is recommended when conservative treatments fail [1,3,4,5].

Patient education constitutes a cornerstone of modern surgical care and is a critical prerequisite for obtaining valid informed consent. Adequate educational interventions not only enhance patient understanding but also contribute significantly to adherence, compliance, and overall treatment satisfaction. Evidence suggests that patients who receive comprehensive and structured medical information are more likely to engage with their treatment plans and follow medical advice over the long term. For instance, in a study evaluating adherence to therapeutic recommendations, 67% of patients who were provided with thorough educational materials and counseling maintained compliance after six months [6]. In the field of hand surgery, targeted patient education programs have been associated with measurable improvements, including reductions in postoperative opioid consumption and enhanced recovery experiences [7].

Hand surgery presents a particular challenge in patient education due to the complexity and density of anatomical structures involved [8]. Conditions such as carpal tunnel syndrome (CTS), which involve the compression of the median nerve within the carpal tunnel, demand a high level of anatomical and procedural understanding from patients to make informed decisions regarding treatment options. Successful management of CTS relies not only on accurate diagnosis and appropriate intervention but also on the patient’s comprehension of pathophysiology, surgical procedures, and potential outcomes. Thus, accessible, accurate, and comprehensible information delivery is especially crucial in this context.

Online patient education materials in orthopedic surgery are consistently written above the recommended grade level [9,10]. A recent study conducted a comprehensive analysis of literature published over the past seven years concerning the readability of patient education materials within the domains of hand surgery and orthopedics [11]. The findings indicated that the average readability of the 77 educational resources evaluated had declined in comparison to data from a prior benchmark study conducted in 2008. These results underscore that, despite increasing awareness, there has been no substantial improvement in the readability of patient education materials in the field of hand surgery. There is, however, a growing consensus within the academic and clinical communities that enhancing the accessibility and linguistic clarity of patient-oriented educational content is critical, and there is a need for innovative and more effective approaches to patient education within this surgical specialty [11,12].

The advent of generative artificial intelligence (AI) tools, such as OpenAI’s ChatGPT, has introduced a novel paradigm for disseminating medical knowledge. These tools can generate real-time, patient-friendly responses to complex medical queries and hold promise for improving access to health information [13]. However, the utility of such AI-driven platforms in the domain of patient education—particularly in the context of CTS—remains largely unexplored. While prior research has evaluated ChatGPT’s capacity to deliver general health information, few studies have assessed the quality, comprehensiveness, and accuracy of AI-generated content from both patient and physician perspectives [13,14].

In the present study, we sought to evaluate the effectiveness of ChatGPT as an adjunctive tool for patient education in carpal tunnel syndrome. Using a designed questionnaire, we systematically assessed the quality of information provided by ChatGPT regarding CTS diagnosis and surgical treatment, as perceived by both patients and healthcare professionals. By analyzing the clarity, completeness, and perceived usefulness of AI-generated educational content, this study aims to contribute to the growing discourse on the integration of artificial intelligence into clinical communication and shared decision-making processes.

## 2. Materials and Methods

In this prospective study conducted at our hand surgery outpatient clinic, all patients presenting with neurologically confirmed carpal tunnel syndrome and a clinical indication for open carpal tunnel release were consecutively enrolled between 1 February 2025 and 28 February 2025 (*N* = 8). Only patients who had not undergone surgery on the opposite side were included. Written informed consent was obtained from all participants prior to inclusion. Following consent, each patient received standardized information regarding carpal tunnel syndrome and its treatment options—specifically surgical decompression via open carpal tunnel release. This information was provided using ChatGPT-4o (OpenAI, San Francisco, CA, USA). To generate the educational content, a prompt was formulated in accordance with OpenAI’s guidelines to elicit an informative and comprehensible explanation of the disease and its management. The specific prompt used was: “Hello. You are now assuming the role of an informative medical professional. A patient presents with neurologically confirmed carpal tunnel syndrome affecting the left/right hand. Surgical intervention via open carpal tunnel release under local anesthesia has been indicated. The procedure is scheduled to be performed on an outpatient basis. Please provide the patient with comprehensive, comprehensible, and medically accurate information regarding the following: an overview of carpal tunnel syndrome, Common causes and contributing risk factors, the rationale, procedure, possible risks and complications associated with the surgery, the expected prognosis, available treatment alternatives, and postoperative care requirements.”

**Figure 1:**
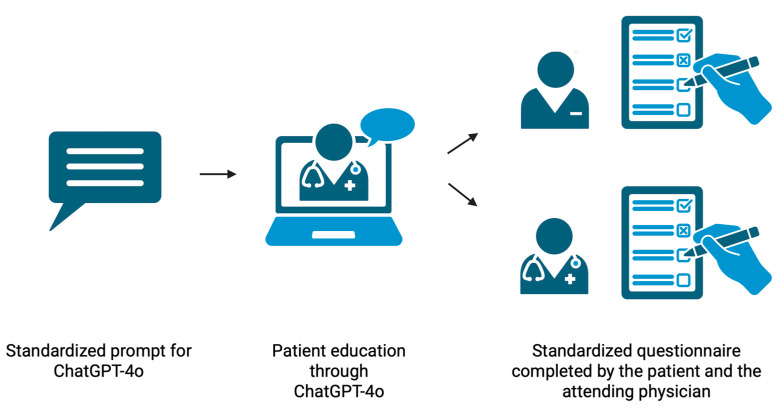
Schematic representation of the workflow.

Afterwards, the patient could ask further questions to ChatGPT, similar to a real consultation. The quality, comprehensibility, and perceived usefulness of the information generated by ChatGPT were subsequently evaluated using a designed questionnaire, which was completed independently by both the patient and the attending physician (Figure 1). It is important to note that the AI-generated information served as a supplemental tool and did not replace the formal physician–patient consultation, which was conducted in full upon completion of the questionnaires. A structured questionnaire was developed to evaluate the quality and completeness of preoperative information provided to both patients and physicians regarding carpal tunnel syndrome and its surgical management. The questionnaire included the following items:

- To what extent were you informed about the causes of the disease?
- How thoroughly were the anatomical foundations of the condition explained?
- How clearly was the surgical procedure described?
- To what extent were alternative treatment modalities presented?
- How comprehensively were the potential risks and complications addressed?
- To what extent were the anticipated benefits and chances of success of the surgery conveyed?
- How well were the long-term consequences of foregoing surgical treatment explained?
- To what extent were postoperative care and recommended patient behavior discussed?
- How would you rate the overall quality of the information provided?
- In your opinion, which relevant aspects were insufficiently addressed?

Both patients and a qualified physician assessed the AI-generated content using a structured questionnaire consisting of twelve items. There were five physicians, with professional levels ranging from residents (*n* = 3) to consultants (*n* = 2). The first nine items were rated using a five-point verbal Likert scale, where “very good” corresponded to a score of 1 and “very poor” to a score of 5; thus, lower scores reflected higher perceived quality. An aggregate score for each question was computed using the following weighted formula:

Overall Score = (“very good”/*N* × 1) + (“good”/*N* × 2) + (“neutral”/*N* × 3) + (“poor”/*N* × 4) + (“very poor”/*N* × 5) (1)

*N* represents the total number of responses per item (*N* = 8 in this study). The final three items consisted of free-text responses requesting key terminology that should be included in a high-quality preoperative educational consultation. An analysis of the completeness of information was conducted through qualitative evaluation of key terms mentioned during the informed consent process. Specific questions were used to assess the presence of critical anatomical and procedural terminology. These included:

- Were all relevant anatomical structures of the carpal tunnel discussed (e.g., ligamentum carpi transversum, osteoligamentous canal, contents of the carpal tunnel)?
- Were all essential components of the surgical technique described (e.g., anesthesia modalities such as local anesthesia, WALANT (wide awake local anesthesia no tourniquet), tourniquet use; surgical steps including incision, dissection, division of the flexor retinaculum, nerve exposure and neurolysis, handling of the thenar branch, inspection and palpation, synovectomy, hemostasis, and wound closure)?
- Were all viable alternative treatment options mentioned (e.g., conservative management, endoscopic decompression, corticosteroid injection)?

The relative frequency of occurrence of each term was calculated as a percentage across all responses to quantify the extent of detail and completeness in the preoperative counselling.

## 3. Results

This study evaluated the quality of preoperative patient information provided by the generative AI model ChatGPT-4o regarding open carpal tunnel release (carpal roof release) in patients with neurologically confirmed carpal tunnel syndrome.

**Figure 2:**
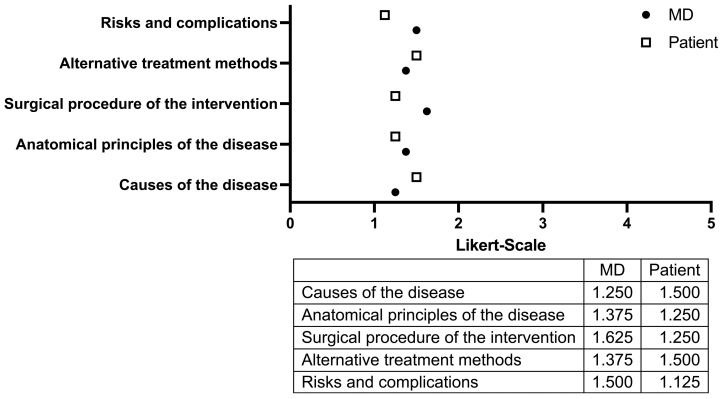
Assessment of the quality of preoperative educational information provided by ChatGPT, evaluated using a verbal five-point Likert scale (1 = very good; 5 = very poor). The informational content was categorized into five domains: etiological factors, anatomical context, surgical technique and procedural details, alternative treatment options, and potential risks and complications. Ratings were independently provided by patients (white boxes) and the consulting physician (black dots).

The first item evaluated the explanation of the disease etiology (Figure 2). Patients rated the explanation between “very good” and “good” with an average score of 1.25. The physician provided a similar but slightly less favorable assessment, rating the content at 1.5. Regarding anatomical explanations, physicians gave a score of 1.25, while patients rated the same content at 1.375. In evaluating the clarity and completeness of the surgical procedure description, patients assigned a score of 1.25. Physicians were more critical, rating the content at 1.625, indicating a perception closer to “good” than “very good.” When assessing the information on alternative treatment modalities, patients assigned a score of 1.5, whereas the physician rated the content slightly better at 1.375. The explanation of procedural risks and potential complications was rated most favorably by patients with a score of 1.125, while the physician gave a rating of 1.5, suggesting slightly less satisfaction.

**Figure 3:**
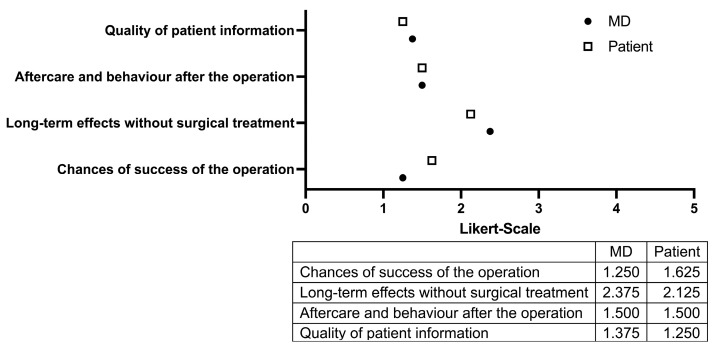
Evaluation of ChatGPT-generated preoperative informal content in the domains of surgical outcome expectations, long-term consequences without intervention, postoperative care, and overall quality. Scores are based on a five-point Likert scale (1 = very good; 5 = very poor). Ratings were independently provided by patients (white boxes) and the consulting physician (black dots).

Regarding the explanation of surgical outcomes and success rates, patients expressed moderate to high satisfaction, assigning a score of 1.625 (Figure 3). In contrast, the physician rated the information more favorably, with a score of 1.25. The poorest ratings across both groups were observed in the domain addressing long-term consequences in the absence of surgical intervention: patients rated this item at 2.125, while the physician evaluated it even less favorably at 2.375. Information on postoperative care and recommended behavioral measures was well received by both groups, with an identical score of 1.5. Overall, the AI-generated content was rated slightly more favorably by patients (mean score: 1.25) than by the physician (mean score: 1.375).

**Figure 4:**
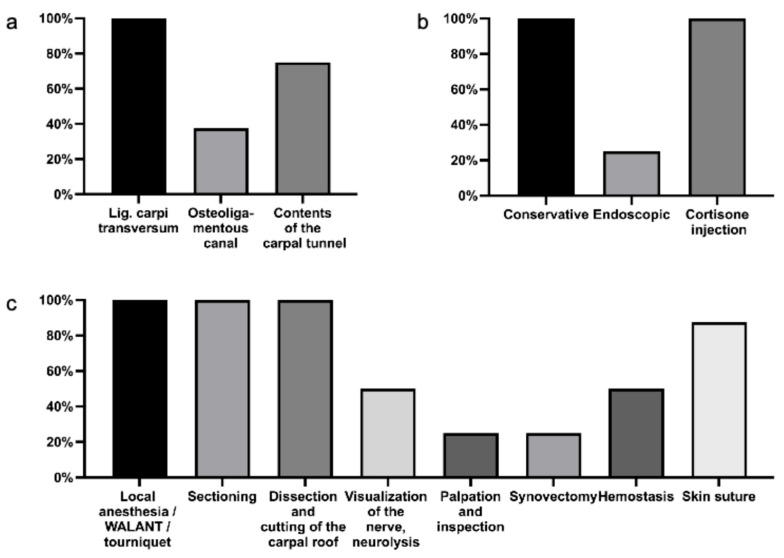
Assessment of the explanatory quality of ChatGPT in the context of preoperative patient education regarding carpal tunnel release. Evaluation was based on the proportion of essential keywords and technical terms appropriately defined and explained, expressed as a percentage. (**a**) Percentage of correctly explained anatomical structures relevant to the carpal tunnel. (**b**) Percentage of alternative treatment modalities mentioned. (**c**) Percentage of adequately described key surgical steps involved in open carpal tunnel release.

The final portion of the analysis assessed the completeness of essential terminology and procedural elements. With regard to anatomical structures, ChatGPT correctly informed patients and physicians about the ligamentum carpi transversum as the “carpal roof” in 100% of cases, described the osteoligamentous canal in 40%, and detailed the contents of the carpal tunnel in 75% (Figure 4a). When addressing alternative treatment modalities, ChatGPT mentioned conservative options and corticosteroid injections in 100% of instances; however, endoscopic decompression was referenced in only 25% of cases (Figure 4b). For the surgical procedure itself, preparatory measures such as local anesthesia, the use of a tourniquet or WALANT technique, skin incision, and release of the carpal roof were all mentioned in 100% of cases (Figure 4c). Key intraoperative steps such as median nerve visualization, neurolysis, and hemostasis were included in 50% of explanations. The identification of residual tightness or synovial hypertrophy and the performance of a synovectomy were noted in 25% of cases. Closure with skin sutures was reported in 80% of the responses.

## 4. Discussion

Preoperative patient education plays a critical role in the surgical pathway. High-quality, comprehensive patient information is essential for informed consent and therapeutic alliance. Studies have demonstrated that preoperative interventions and patient education exert a significant influence on postoperative outcomes following elective hand surgery [15]. However, the time constraints of routine clinical practice often limit the depth and consistency of preoperative discussions. In this context, we developed a tailored prompt for ChatGPT-4o to deliver standardized educational content regarding carpal tunnel syndrome and its surgical treatment via open carpal tunnel release.

In the present study, we assessed the quality of information generated by ChatGPT from both the patient and physician perspectives using a structured and validated questionnaire. Overall, the findings demonstrate that both groups were predominantly satisfied to very satisfied with the content provided. This showed that the idea of using ChatGPT to perform complete and conversational patient education is promising. The small number of patients included in our study is a major limitation. However, multicenter and multilingual studies on a large cohort are required to generate further evidence.

The etiology of CTS and the associated procedural risks and complications were communicated at a consistently high standard. The anatomical background of the syndrome was generally well conveyed, although certain key anatomical details—such as the osteoligamentous canal and the full contents of the carpal tunnel—were not consistently addressed. The surgical procedure itself was described in a satisfactory manner according to both patients and physicians. Nevertheless, some important intraoperative concepts—including neurolysis of the median nerve, synovectomy, palpation for residual constrictive structures, and hemostasis—were either underrepresented or absent. Additionally, while ChatGPT reliably referenced conservative treatment modalities and corticosteroid injections, it failed to mention endoscopic release as a valid surgical alternative, thereby limiting the comprehensiveness of the information. Notably, the explanation of surgical prognosis and the potential long-term consequences of non-treatment were rated as average or neutral by some respondents. Postoperative care and behavioral recommendations were generally presented at an acceptable level, though without nuanced individualization. These findings suggest that ChatGPT may serve as a valuable tool in the preoperative education and counselling of patients with carpal tunnel syndrome.

Looking ahead, generative AI tools like ChatGPT hold promise as adjunctive resources in patient education, particularly by delivering standardized, comprehensible information and addressing preliminary queries. Their application in hand surgery has already been explored. Gezer et al. evaluated ChatGPT’s responses to questions about trigger finger, finding the model generally reliable but emphasizing the need for expert oversight before integrating it into patient education [16]. Similarly, White et al. assessed ChatGPT 4.0’s responses to FAQs about boxers’ fractures and found them to range from adequate to comprehensive, supporting its potential as a supplementary communication tool [17]. In our study, we found that the information provided by ChatGPT regarding carpal tunnel syndrome was associated with a high level of patient satisfaction. However, important aspects such as the long-term consequences of untreated disease, the detailed steps of the surgical procedure, and the endoscopic surgical alternative were insufficiently addressed. Nonetheless, patient engagement with such technologies remains limited. A study surveying 511 hand surgery patients revealed that only 3.9% used chatbots regularly, while 70% had never interacted with one—raising concerns about patient readiness to adopt AI-driven tools [18]. Moreover, a German RCT involving 261 patients showed that while 98% felt well-informed post-consultation, 24% could not recall potential complications, suggesting gaps between perceived understanding and actual comprehension [19]. Crucially, AI tools lack the capacity to interpret non-verbal cues or respond to emotional concerns—integral elements of preoperative discussions. Therefore, while AI may enhance educational outreach, it cannot substitute the trust and nuance inherent in face-to-face consultations.

Recent studies have further explored the clarity, accuracy, and acceptance of ChatGPT-generated educational content compared to traditional sources. Gao et al. found online materials on carpal tunnel release often lacked readability and actionability [20], while a comparative study involving ChatGPT, WebMD, and the Mayo Clinic revealed no significant differences in readability or patient preference [21]. However, younger patients showed greater trust in institutional platforms, suggesting generational divides in AI acceptance [21]. Another study comparing ChatGPT 3.5 with top Google results for carpal tunnel syndrome found ChatGPT’s responses equally accurate but more concise [22]. Our findings largely corroborate this observation. Both physicians and patients rated the overall quality of the AI-generated information provided by ChatGPT highly, with mean scores ranging from 1.25 to 1.375. In plastic surgery, Zhang et al. demonstrated that ChatGPT improved the clarity of postoperative instructions for lay audiences, enhancing compliance, although physicians noted persistent limitations in depth and contextual nuance [23]. A French study assessing ChatGPT-generated materials for hand surgery found non-medical users rated them positively, while surgeons favored content created by experienced educators, especially for visual aids [24]. Within carpal tunnel syndrome, ChatGPT showed promise in providing accessible, validated information [25]. However, its limitations—such as outdated references, hallucinated citations, and lack of access to recent literature (e.g., ultrasound diagnostics) [26]—remain critical concerns. Moreover, version-specific variability complicates generalization of results.

Beyond hand surgery, evaluations across specialties—including sleep apnea, thyroid nodules, periodontology, and nuclear medicine—have affirmed ChatGPT’s general appropriateness in delivering medical content, while also identifying issues in readability and technical precision [13,14,27,28,29]. Despite prompt engineering efforts, most content exceeded the recommended grade level for patient materials [13,14,30]. Additionally, AI-generated responses can omit essential information or introduce inaccuracies, necessitating expert validation [27,28]. Hallucinations—where AI models fabricate false data—pose a significant risk [31]. The legal and ethical implications of AI deployment further complicate clinical integration. Within the European Union, critical regulatory frameworks include the General Data Protection Regulation (GDPR), the forthcoming Artificial Intelligence Act, and the Medical Device Regulation. These laws address not only compliance and liability, but also public trust in both AI systems and physicians, underscoring the ethical complexity of AI integration into healthcare communication.

This study has several limitations. The small sample size restricts the generalizability of the results, though this reflects its nature as a pilot study. The use of questionnaires limits the depth of quality assessment, as responses depend on both question design and the nature of interaction between patients or physicians and ChatGPT. Additionally, participants were informed about the study in advance, and no blinding was employed. Finally, the lack of data on participants’ cultural backgrounds may have influenced outcomes.

## 5. Conclusions

ChatGPT offers valuable support for patient education in carpal tunnel syndrome, particularly by supplementing physician consultations and enhancing understanding. It may help standardize preoperative information and clarify medical terms, especially when clinical time is limited. However, it cannot replace direct physician communication due to its lack of personalization, emotional awareness, and current concerns regarding data privacy, accuracy, and medico-legal implications.

## Data Availability

The data presented in this study are available on request from the corresponding author.
